# IoT System Selection as a Fuzzy Multi-Criteria Problem

**DOI:** 10.3390/s22114110

**Published:** 2022-05-28

**Authors:** Galina Ilieva, Tania Yankova

**Affiliations:** Department of Management and Quantitative Methods in Economics, University of Plovdiv Paisii Hilendarski, 4000 Plovdiv, Bulgaria; tsvl@uni-plovdiv.bg

**Keywords:** IoT, Agriculture 4.0, MCDM, MABAC method, intuitionistic fuzzy sets, distance measure

## Abstract

This research aims to analyse the applications of IoT in agriculture and to compare the most widely used IoT platforms. The problem of determining the most appropriate IoT system depends on many factors, often expressed by incomplete and uncertain estimates. In order to find a feasible decision, this study develops a multi-criteria framework for IoT solution selection in a fuzzy environment. In the proposed framework, a new modification of the Multi-Attribute Border approximation Area Comparison (MABAC) method with a specific distance measure via intuitionistic fuzzy values has been presented as a decision analysis method. The new technique is more precise than existing crisp and fuzzy analogues, as it includes the three components of intuitionistic numbers (degree of membership, degree of non-membership and hesitancy degree) and the relationships between them. The effectiveness of the new decision-making framework has been verified through an illustrative example of ranking IoT platforms.

## 1. Introduction

The rise of information technologies and the expansion of the Internet have transformed traditional machines and devices in part of a new Internet of Things (IoT) economy, gaining ground across many industrial sectors. Over the past two years, the outbreak of COVID-19 has aggravated food security and exacerbated the problem of digital transformation in agriculture. Agriculture 4.0, IoT in agriculture and smart agriculture are synonyms of technological innovation that has fundamentally change the business processes in the agricultural value chain [1]. The Internet connects sensors, robots, agricultural machinery, analytical tools and farmers in a new and inventive way. The deployment of digital technologies, such as IoT, big data and machine learning (ML) boost revenues from the land, greenhouses, warehouses and food processing plants [2].

Augmented reality is an important direction for the digital transformation of agricultural systems. It enriches the capabilities of field checking and the early detection of risk of crop failure and thus speeds up operations and increases yields [3]. Through enhanced monitoring and control, a “digital twin” of an agricultural system could be created. The system’s efficiency is optimized by simulating the real-time behaviour of physical assets (plants, harvest, machinery and personnel). The advantages of virtual copies of agricultural objects and business processes in comparison with classical simulation models are scenario management and instantaneous verification in a real-world context. Traversing the whole range of possible changes of fields’ parameters (light, water, nutrients, humidity and temperature) of digital copies improves harvest quality and extends the post-harvest durability of grains, vegetables, fruits and nuts [4].

The incorporation of blockchain into IoT platforms is also useful for farmers, suppliers and consumers, as this combination guarantees the quality of production from the field to the table. Blockchain ensures the security of data and operations in a supply chain through cryptographic algorithms [5,6]. The fifth generation of mobile telecommunications (5G) allows for lower latency, which means a faster exchange of big data and a shorter response time of web-based software. Sensors become more responsive, and farmers react to changes in the field in real time [7]. Predictive maintenance and the remote and centralized control of operations are some examples of 5G applications in agriculture [8].

During the COVID-19 pandemic, the preventative measures (blocking, restrictions on the movement of goods and people, social distancing, curfew and restrictions on large gatherings) have revealed weaknesses in food supply chains and have called into question the state of food security and farmers’ incomes. The pandemic has revealed the need for sustainable and efficient agricultural value chains [9]. In the case of unforeseen circumstances, digital farming tools facilitate the transformation of the food industry and improves its flexibility and resilience. The advantage of IoT in this crisis situation is that it optimizes supply chains, reduce costs and minimizes response time [10].

The benefits of connecting physical objects in farms to the Internet are as follows:

- Saving resources (time and labour) by remotely monitoring environmental and infrastructural conditions instead of on-site inspections;
- Increasing the efficiency and sustainability of production with traceability, timeliness and the security of the food supply and reduced waste;
- Improving decision-making even in the case of abrupt changes in market demand through real-time data analysis of the entire value chain.

The decision-making process for IoT system selection could be supported by methods taking into account many factors in their calculations. According to recent studies, three main approaches have been developed: mathematical programming (optimization) [11,12,13], ML [14] and multi-criteria decision making (MCDM) methods [15,16,17,18,19,20,21,22,23,24,25,26].

Optimization models often indicate only local optimums instead of the global solution. A disadvantage of ML methods is that the quality of their solutions depends on dataset quality. In contrast, MCDM methods work well even on small datasets; dozens of methods have been developed for determining object rankings and weights of attributes and their combinations; the obtained results are easily interpreted.

Each MCDM model consists of three main elements: a finite set of alternatives, at least two criteria and at least one decision-making method with crisp or fuzzy estimates and criteria weights. As MCDM has been an active area of research in recent years, decision analysis methods have found applications to solve the IoT system selection problem.

Some researchers have proposed referential models for IoT platform selection, including one or more existing MCDM methods; for example, Simple Additive Weight (SAW), Technique for Order of Preference by Similarity to Ideal Solution (TOPSIS) and VIseKriterijumska Optimizacija I Kompromisno Resenje (VIKOR) [17], a set of MCDM methods [18] and the Decision-Making Trial and Evaluation Laboratory (DEMATEL) to determine the cause–effect relationship of predefined criteria [19].

Another segment of studies has suggested new hybrid models combining a method for determining the importance of criteria weights with a multi-criteria method. For example, Mashal et al. have built a meta-model combining Analytical Hierarchy Process (AHP) and SAW [20], Youssef—Best Worst Method (BWM) and TOPSIS [21]. The Silva and Jardim–Goncalves model has combined AHP and Elimination and Choice Expressing Reality (ELECTRE) [22].

As the estimates of compared IoT platforms are often inaccurate and uncertain, a mix of multi-criteria decision-making and fuzzy set theory could be employed to rank them [23]. Lin et al. have introduced the probabilistic linguistic term sets (PLTSs) to express the users’ preferences of IoT platforms. Then, the authors introduced a new combination between BWM and PLTSs for the calculation of the criteria’s importance. After that, a new modification of Interactive and Multi-criteria Decision Making (TODIM—according to the method’s name in Portuguese) in probabilistic linguistic values has been proposed to rank IoT platforms [15].

Kondratenko et al. have built a Mamdani inference model for the comparison of specialized IoT systems. The most important criteria that determine the priority of compared platforms are reliability, dependability, safety and security [24].

Ilieva et al. have presented a new methodology combining several MCDM methods for cloud service choice in a fuzzy environment. According to this study, service functionalities, customer support and rating and security are the most important factors influencing cloud systems evaluation. To demonstrate the effectiveness of the pro-posed procedure, the authors have solved the problem of cloud storage analysis using the Measurement of Alternatives and Ranking according to the Compromise Solution (MARCOS) method via triangular fuzzy numbers [25].

Chakraborty et al. have evaluated nine cloud service providers using the Distance-Based Approach (DBA) with triangular fuzzy values of fourteen evaluation criteria (grouped as quality, technological and economic factors) [26].

Although there have been many studies recently concerning the IoT system selection problem, there is no universal approach or unified procedure for its solution. The studies described above provide some insights on the comparison of IoT systems, but they do also demonstrate some drawbacks:

(1) Often, criteria systems include only few technical characteristics of IoT systems; however, evaluation should also be a function of many other factors; for example, social and organizational parameters;
(2) If IoT system assessment depends on qualitative factors, then their estimates are often subjective and should be made by a team of experts and fuzzy numbers;
(3) The majority of the proposed fuzzy multi-criteria solutions implement only one or two MCDM methods.

To overcome these shortcomings, in this manuscript we propose a new fuzzy methodology for IoT solutions’ evaluation. The selection of an appropriate IoT system according to the needs of a given agricultural company is a complex procedure, depending on many factors. The group multi-criteria approach has the potential to solve this multi-factor problem. Alternatives include a variety of IoT (general purpose or specialized) systems available on the market. The solution to the problem is the system that best meets the preferences of a particular farm.

The aim of this study was to create a methodological framework for the multi-criteria management of the procedure for the selection of an IoT system in a fuzzy environment with a focus on agriculture companies.

The tasks of this study are as follows:

(1) present the most widely used IoT platforms;
(2) explore the impact of multi sensors IoT systems on agricultural companies;
(3) develop a conceptual framework for the multi-criteria analysis of an IoT system;
(4) create a modification of Multi-Attribute Border approximation Area Comparison (MABAC) for an intuitionistic fuzzy environment;
(5) verify the proposed framework and the new method through a practical example.

The limitations of our study are as follows:

1. The problem of finding the most suitable IoT system can be solved using different approaches, but our study is limited to the decision analysis methods.
2. In order to make an informed decision, decision-makers need to have relevant information (expert opinions, literature sources, decision rules, etc.) for the preliminary assessment of the need for an IoT system, the formulation of user requirements and the calculation of weighting coefficients and comparison index.
3. In the new framework, only IoT systems for which the necessary information (features, reviews, comparisons, etc.) to build a decision matrix is provided can be evaluated.

The rest of this article is organized as follows: In Section 2, the peculiarities of the most widely used general-purpose and specialized IoT platforms for Agriculture 4.0 are analysed. Next, the previous studies on the topic of multi-criteria selection of IoT systems are summarized. Section 3 presents the new multi-criteria decision making (MCDM) framework for IoT system selection and a modification of the MABAC method with uncertainty in estimates of decision makers. In the next section, the problem of IoT product selection is solved using the proposed methodology. The last section summarizes the obtained results and outlines directions for future work in enhancing the multi-criteria methods and expanding their applications.

## 2. IoT Technology and Its Application in Agriculture

Smart farming represents the applications of IoT through a variety of autonomous devices connected to each other via the global network. By using intelligent sensors, drones, robots and agricultural machinery, farmers inspect and supervise the agricultural processes, from planting, growing and dispatching to the storage and distribution of crops [27,28].

However, managers and other stakeholders in agribusiness are still not familiar with the capabilities of IoT technology in the remote monitoring of a huge amount of data and controlling physical objects in real-time without human interference. In the next section, the basic characteristics of IoT technology are presented. Then, the most widely used IoT systems are compared and applications of IoT in agriculture are analysed.

### 2.1. IoT Basics

According to the NIST definition, IoT is a network of devices that contains hardware, software, firmware and actuators which allows the devices to connect, interact and freely exchange data and information [29,30,31]. This network encompasses computing devices, mechanical machines and various other physical objects that have unique identifiers and exchange data, even without human intervention [32,33,34].

During the fourth revolution in agriculture, farms must adopt new digital technologies in order to be competitive. However, the digital transformation through IoT poses three main technological and business challenges.

The first one is the growing demand for connectivity. The proliferation of 5G stimulates the need for communication between an increasing number of sensors. A possible solution to the server overload problem is the deployment of blockchain storage in decentralized computer networks.

The next challenge is related to device security and data privacy. The threat of unauthorized access to data or the replacement of a device’s instructions requires manufacturers to continuously improve IoT protection and reliability [35].

The third challenge is the lack of standards unifying the requirement specifications in the manufacturing of IoT components. The multitude of devices from different manufacturers using various communication protocols hinders the portability, interoperability and manageability of IoT systems. Several standardizing bodies have already established some important standards for seamless communication between different IoT systems and among entities within an IoT system [36]. Unfortunately, the harmonization has not yet been completed, which complicates the IoT development process and increases its cost.

IoT technology connects a variety of objects to the Internet, uniting the virtual and physical worlds. This merging of the two worlds is a prerequisite for the more efficient usage of available resources. IoT minimizes human efforts because devices can connect and communicate directly with each other and perform various tasks autonomously. Existing security, data protection and interoperability challenges can be resolved through the active engagement of stakeholders.

### 2.2. The Peculiarities of Major IoT Platforms

From a cloud technology point of view, IoTs platforms connect devices and applications within a cloud ecosystem. They are also known as IoT Platforms as a Service (PaaS), i.e., by using these platforms, users create their own applications and services employing built-in IoT features. An IoT application usually includes the connecting of remote devices through the cloud, collecting data from devices, remote control, analysis and visualization and the management of application versions and user access rights. This section briefly describes the main features of the most widely used IoT platforms (AWS IoT Core, MS Azure IoT, Google Cloud IoT Core, IBM Watson IoT and Oracle IoT Service).

AWS IoT Core (https://aws.amazon.com/iot/, accessed on 1 March 2022) is a managed cloud service that connects IoT devices and interacts with cloud applications and other devices. The platform supports a large number of devices, processes and messages, and routes those messages reliably and securely. AWS IoT users create their own solutions with a combination of over 60 AWS cloud and edge services. Common service elements across all deployment models are AWS IoT Greengrass, Amazon Monitron and AWS Panorama. AWS also offers a portfolio of capabilities for edge-centric computing that includes intelligence and inference closer to monitored events. Unfortunately, edge cloud and on-premises deployment models offer fewer capabilities, especially relating to data management and analytics [37].

MS Azure IoT (https://azure.microsoft.com/en-us/overview/iot/, accessed on 1 March 2022) is a collection of Microsoft-managed cloud services that connect, monitor, and control IoT assets. Each IoT solution is made up of one or more IoT devices that communicate with one or more back-end services hosted in the cloud. Azure IoT employs Azure platform and offers cloud, edge and hybrid deployment options. Azure IoT services can run on-premises and Azure IoT edge solutions offer architecturally secure isolation through a hierarchical deployment approach. Customers have access to the Microsoft products and a marketplace for third-party software. Azure IoT can be used in many vertical markets such as healthcare, retail and manufacturing [37].

Google Cloud IoT Core (https://cloud.google.com/solutions/iot, accessed on 1 March 2022) is a cloud service consisting of two main components: a device manager and protocol bridges. The former registers devices with the service, so the user can monitor and configure them, while the latter can be employed by devices to connect to Google Cloud Platform. Typical use cases include asset tracking, visual inspection and quality control in retail, smart parking, transportation and logistics.

IBM Watson IoT (https://cloud.ibm.com/docs/IoT?topic=IoT-iot-cloud-index, accessed on 1 March 2022) pro-vides application enablement, data and device management, analytics, integration and security. This platform is delivered as a collection of services with the IBM Cloud or can be deployed on-premises via private cloud capabilities. It is deployed in manufacturing, utilities, transportation and logistics. Common use cases are predictive maintenance and asset monitoring. Customers use the Node-RED open-source programming language to personalize and extend the functionality of Watson IoT [38].

Oracle IoT Cloud Service (https://docs.oracle.com/en/cloud/paas/iot-cloud/, accessed on 1 March 2022) can be integrated with Oracle’s middleware solutions and enterprise applications. Oracle’s industrial IoT (IIoT) are suitable for production, asset and fleet and service monitoring. A typical solution comprises data management capabilities, analytics, user interfaces with custom dashboards and built-in integrations with manufacturing, maintenance and asset management systems. These solutions can run on Oracle Cloud but are available in an on-premises version via private cloud [38].

The five above-mentioned IoT systems share some common characteristics. They are designed to be platforms with general purpose and, therefore, they are best suitable for standardized solutions. A disadvantage of these platforms is that they are oriented entirely to the cloud ecosystem of their vendor. As a result, their work processes are more complex, and their settings require specialized knowledge about the particular cloud infrastructure (especially in the AWS case). These ecosystems include predefined modules and applications for a variety of IoT use cases. However, their main disadvantage is the lack of personalization.

Table 1 summarizes some main features of the presented IoT platforms. These attributes can be built into an evaluation system for the selection of an IoT platform and its components.

IoT Core Functions: The compared IoT platforms gather and store data from and communicate with disparate IoT endpoints in a secure way. They also employ analytics to find hidden patterns in the data and provide applications that enable organizations to visualize and take action on the insights extracted from the data. The five vendors offer a cloud-edge hybrid model, allowing for a consistent experience on both the edge and the cloud.

Edge Computing Solutions: Edge computing minimizes the latency of cloud communication between the client and server by bringing the infrastructure closer to the ends of the network. With edge computing, the compared platforms exchange data with less delay and lower cloud server bandwidth usage. Customers can gather edge data and send it to the IoT Cloud via SDK for devices, gateway software or cloud-based IoT connectors that can do protocol translation and then send messages to the cloud services.

Communication Protocols: Google Cloud IoT, IBM Watson IoT and Oracle IoT employ only two data protocols: HTTP and Message Queue Telemetry Transport (MQTT). HTTP protocol is not preferred for IoT data transfer because of its high cost, low battery life, high power consumption and weight issues. In contrast, MQTT is a lightweight IoT data protocol, but it does not support a defined data representation and device management structure mode. As a result, the implementation of data and device management capabilities is entirely platform specific. In addition to these two protocols, AWS IoT and Azure IoT also support WebSocket (in Azure IoT case via Advanced Message Queuing Protocol—AMQP). WebSocket is an appropriate choice for IoT networks where data is bidirectional communicated continuously across multiple devices. AMQP, as an open standard application layer protocol, is used for transactional messages between servers.

Data Processing and Analytics: IoT platforms offer the analytical services of the respective vendor’s ecosystem. The advantage of built-in analytics tools is that users train, deploy and manage their new AI models, and then prepare data and analyze information in a single integrated environment. Unfortunately, these modules require payment to all of the above-mentioned platforms.

Each of the presented platforms is the leader on vertical markets in several industries. For example, AWS IoT is the most suitable for smart home deployment, while Microsoft Azure IoT demonstrates high efficiency on the Internet of Medical Things (IoMT) market. Google Cloud IoT brings the most value to transport companies. IBM Watson IoT and Oracle IoT are leaders in the management of large enterprises with complex infrastructure and a large number of sensors, as both companies have experience in the management of industrial equipment.

The optimal selection of an appropriate IoT system should not be limited to the most widely used platforms and the indicators for their comparison in Table 1. There are a variety of open-source and specialized IoT alternatives, some of which even outperform the aforementioned technological leaders in some industries.

### 2.3. IoT for Agricultural Activities

In traditional agriculture, due to the heterogeneity of risks and long production cycle, revenues are uncertain. The concept of intelligent agriculture is revolutionizing the sector. IoT systems track the dynamic changes in farms (meteorological conditions, soil quality, availability and cost of labor force), forecast the potential risks of crop damage, plan activities in advance and take preventive measures for protection in case of emergencies. Applications of IoT technology increase the yield and decrease the costs of agricultural production without unnecessary treatment. Agriculture is no longer seen as a high-risk and labor-intensive industry because the harvest is predictable, and labor productivity increases and the quality of products in the food supply chain are guaranteed [39,40]. Although IoT is still not a very popular technology in agriculture, its market is constantly growing. The size of the global market for intelligent agriculture is expected to quadruple by 2026, reaching $22.5 billion (compared to just over $5 billion in 2016) [41].

**Figure 1:**
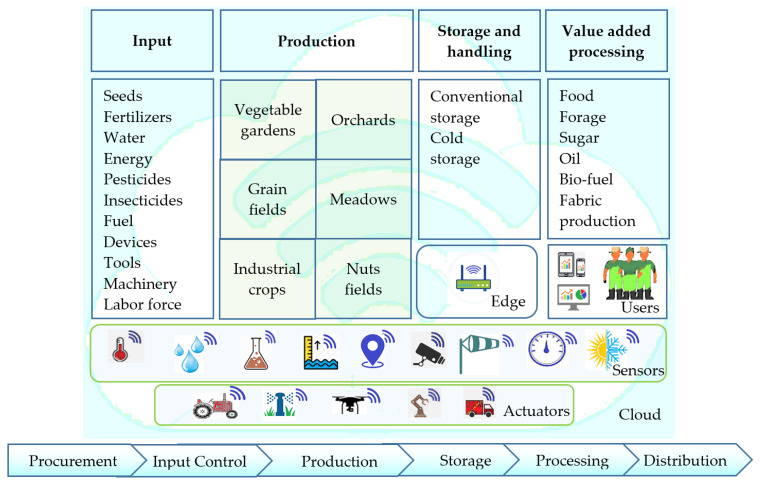
IoT in the agricultural supply chain.

In this section, we discuss the main application areas of IoT in the agricultural supply chain. This supply chain includes of a variety of participants—input suppliers (cooperatives, agro-dealers), farmers (growers), processors, shipping companies, wholesalers, retailers and final consumers. The digitization of agricultural supply chain, supported by IoT technology, is depicted in Figure 1. Every business process, performed between actors along the crop trajectory, could be empowered by the use of IoT technology, including ploughing, sowing, irrigation, weeding and harvesting. Each product follows its own path to reach customers, and every relationship between participants (supplier-farmer, farmer-processing company and farmer-distributor) is assisted by IoT devices. The following sections consider the applications of IoT in agriculture through business processes: supply of materials, crop management and distribution of food products.

- Supply of agricultural materials

IoT technology improves the traceability of agricultural materials (seeds, minerals, fertilizers, pesticides, fuel, and water) throughout the crop life cycle. IoT devices track quality, location and time during materials’ transfer from suppliers to warehouses or fields. To this end, IoT platforms are equipped with various data readers: intelligent tags (barcodes, near field communication, RFID or low-power Bluetooth), which are set up by using a tagging inventory system. For the supply department, IoT ensures accountability of costs and timely delivery with optimal routes, schedules and balanced vehicle loads. The accurate prediction of required quantities reduces excess inventory and improves the efficiency of contract and cost management [42,43].

- Crop management

Through IoT platforms, farmers observe and manage agricultural processes such as planting, growing, harvesting and harvest management via connected sensors installed in fields or greenhouses. Agricultural companies also apply IoT for better management of crop irrigation. The connected sensors can monitor the level of precipitation and humidity and automatically adjust the irrigation schedules (precision irrigation). Data on soil chemical composition and plant growth facilitates the selection of the most appropriate combination of fertilizers and their amount. As a result, the crop yield conditions are optimized and water, energy and fertilizer consumption are reduced [44].

Sensors warn farmers about pests and diseases immediately preventing their spread and minimizing damages [45]. Robots and machinery in agricultural fields operate without downtime and thus reduce risks farmers face such as excessive rainfalls, floods, droughts, freezing or toxic fertilizers. Drones are also useful in farming because they transmit real-time video and even spray various pesticides and fertilizers [46]. Greenhouses also deploy multi-sensor systems that monitor facility parameters and plant growth and automatically adjust equipment to provide the most appropriate conditions for each plant.

The conventional monitoring of crops in storage requires a lot of time and resources. Even in the case of storage equipped with wired sensors, operators should check the storage conditions on-site, which is a continuous and laborious process. IoT systems allow more than remote sensing and notifications. The collected historical data reveal trends regarding harvest states and offer decisions to change the method of storage or sale [47,48].

- Agricultural distribution

IoT systems keep track of the movement of crops to processing plants, distributors’ warehouses and consumers. Sensors in transport containers collect and transmit data about the route, location and status of the shipment in real time. In this way, agricultural companies increase their efficiency, speed up delivery time and improve customer service [49,50].

In agriculture, IoT redesigns business processes (preparation of soil, sowing, adding fertilizers, irrigation, harvesting and storage) to protect crops and optimize yield. The main advantages of IoT applications in agriculture are as follows:

(1) IoT devices and analytics guarantee timely field operations under cost control;
(2) Risk in terms of quality and quantity of food supply and safety is reduced and, thus, the relationships between agricultural producers and consumers are strengthened;
(3) The agricultural supply chain becomes efficient and sustainable.

The stability of the IoT-based model of an agricultural system depends on its completeness and accuracy. The proposed approach complicates agricultural supply chain but increases its stability. Implementing the model also helps stakeholders to predict the long-term consequences of their actions and thus further bolster food security, increase revenues and enhance environmental sustainability.

Using IoT technology, agricultural and processing companies can build a reliable interorganizational information system integrating data collection, business process modeling and planning between supply chain participants for better communication and coherent decision-making.

### 2.4. IoT Solutions in Farming

In this section, we present some basic features of the most widely used IoT systems in agriculture.

Actility (Paris, France, 2010, https://www.actility.com/precision-agriculture, accessed on 1 March 2022) provides detection, monitoring and control over long distance (over 15 km) of a wide variety of key agricultural data: soil temperature and moisture; weather, rainfall and water quality; airborne pollution; crop growth; smart connected harvesters and irrigation equipment; fire, theft and flood detection.

Arable (San Francisco, CA, USA, 2014, https://www.arable.com, accessed on 1 March 2022) sets up a new generation of IoT tools that enable farmers to take advantage of advanced sensors, wireless networks and machine learning recommendations to improve crop growth via agronomic models and intuitive interface.

CropX (Netanya, Israel, 2013, https://cropx.com, accessed on 1 March 2022) employs IoT technology to analyse soil data and integrate it with crop models, satellite imagery and weather forecasts to help farmers cut crop-input costs by driving water, fertilizer, energy and labour savings.

Crop Performance (San Francisco, CA, USA, 2009, https://crop-performance.com, accessed on 1 March 2022) provides geospatial analytics enabling growers and food supply chain to increase crop yields, conserve resources and monitor the ecological impact of growing safe and healthy food. Crop performance analytics create supply decisions based upon crop yields prediction in advance of the harvest.

Cropwise Operations (former Cropio) (Zurich, Switzerland, 2014, (https://www.cropwise.com/operations, accessed on 1 March 2022) as a decision-making tool aims to optimize fertilization and irrigation, and thus reduces the amount of fertilizer and water used. Cropwise Operations, combining weather information and satellite data, also makes it possible to monitor crops and yield forecasts.

Farmapp (Leopold, Australia, 2014, https://farmappweb.com, accessed on 1 March 2022) is an Integrated Pest Management (IPM) software-based service for crops. The software includes a combination of scouting and fumigation applications with sensors and brings IoT devices to the agricultural sector. Using soil sensors, spraying applications and weather stations, growers are able to receive up-to-date data regarding growing conditions.

Growlink (Denver, CO, USA, 2015, https://www.growlink.com, accessed on 1 March 2022) uses crop scheduling, labour forecasting, compliance logging and reporting and task management. Controllers and application give users remote access to monitor sensor data in real time and control any connected devices using a mobile phone or computer. Users can add an unlimited number of power block outlets to switch on or off any hardware such as ventilation blowers, pumps, lights, dehumidifiers, humidifiers, water chillers, heaters, fans or any other electrical device.

Kaa (Miami Beach, FL, USA, 2017, https://www.kaaiot.com/use-cases/smart-farming, accessed on 1 March 2022) is a versatile IoT platform that can be used to create a broad variety of IoT applications, including those used for smart farming. There is no need for coding or previous IoT development experience to use this platform.

Mothive (London, UK, 2015, http://www.terraprimagroup.co.uk, accessed on 1 March 2022) designs IoT solutions to collect environmental and situational awareness data from any location and transform it into knowledge to support customers using trigger alerts and automatic responses. After crops, soil and weather data are processed and correlated with weather and satellite data, the system enables the prediction of crop yields and diseases and improves resource usage and site management through a live alert system.

Particle (San Francisco, US, 2012, https://www.particle.io/agriculture, accessed on 1 March 2022) creates edge-to-cloud IoT development tools including development kits, production modules and asset tracking teams. Particle offers different IoT solutions for connectivity, hardware, device cloud and applications. For better connectivity reasons, it offers three main products: Wi-Fi, Cellular and Mesh.

Pycno (London, UK, 2014, https://pycno.co/platform, accessed on 1 March 2022) is bringing continuous data monitoring and system control to agricultural farms. The wireless sensors establish a simple and low-cost way to collect real-time weather or soil data from a field or greenhouse, visualise it using cloud-driven analytics and drive different control systems.

Rayven (Sydney, Australia, 2016, https://www.rayven.io/industry-solutions/agriculture-farming-iot/, accessed on 1 March 2022) provides organizations with fast, affordable access to IoT, ML and real-time analytics solutions via a drag-and-drop, codeless platform. The platform combines industrial data science and deployment services to gather real-time insights and achieve business goals.

Semios (Vancouver, BC, Canada, 2010, https://semios.com, accessed on 1 March 2022) is a scalable, data analytics platform for growers that helps predict, identify and prevent pest and disease pressure. Providing updates every 10 min, the platform applies big data analytics and ML to reduce and mitigate crop risks for growers.

SoilScout (Helsinki, Finland, 2013, https://soilscout.com/applications/agriculture, accessed on 1 March 2022) optimizes water and energy usage by guaranteeing permanent wireless monitoring. SoilScout wirelessly provides critical insight into data from deep below the surface. Farmers and agriculture professionals employ SoilScout to understand their fields, optimize soil conditions for better growth and improve crop production, also reducing operational costs and water consumption (https://www.linkedin.com/company/soil-scout/about/, accessed on 1 March 2022)

ThingWorx (Boston, MA, USA, 2012, https://www.ptc.com/products/iot, accessed on 1 March 2022), a PTC Technology product, is an IoT and augmented reality platform for industrial enterprises. The platform connects devices and systems, normalizes and analyses data, implements applications and user interfaces, manages and remotely controls devices and delivers new types of experiences through technologies like augmented reality.

Depending on their purpose, the IoT systems described above can be divided into two main groups:

(1) IoT platforms for application development—Actility, Kaa, Particle, Rayven, Cropwise Operations and ThingWorx;
(2) IoT systems (intelligent sensors and analytical platform)—Arable, CropX, CropPerformance, Farmapp, Growlink, Mothve, Pucho, Semios and SoilScout.

Some IoT products are industry independent, while others are specifically intended for agriculture; for example, Farmapp and Growlink—greenhouses automation; Arable, CropX, CropPerformance, Mothive, Semios and SoilScout—crop management. The majority of agricultural IoT software is proprietary, while some IoT platforms are open source (for example, Kaa and Particle).

IoT technology has various applications in smart agriculture. It overcomes the dependence on human labor and meteorological conditions by automating processes such as fertilization and pest control. Intelligent sensors and devices track the entire lifecycle of plants, increasing yields through more efficient use of resources (precise irrigation, control of soil quality, rapid detection of growth abnormalities and local treatment with pesticides). Implementing IoT systems, farmers deliver products with better quality at a lower price.

Despite the many benefits of IoT, this technology has some disadvantages; for example, planning, building and managing such a system is often a complicated project that could be implemented only by experts with a variety of IT skill sets and expertise such as IoT, data analysis and web development.

## 3. Methodological Framework for IoT Platforms Evaluation

In this section, we introduce the basic components of multi-criteria decision-making theory, formulate the selection of IoT system as a MCDM problem, present new theoretical framework for reliable ranking of alternatives and propose a new MABAC modification for multi-criteria decision making.

### 3.1. Main Components of MCDM Theory

The goal of MCDM (multi-criteria analysis) is to determine the relative significances or priorities of a set of *N* alternatives according to a given set of *M* criteria (attributes). Some of the criteria can be maximizing (beneficial), while others can be minimizing (cost). The solution of the problem of multi-criteria analysis consists of two stages: (1) describing the alternatives and evaluation criteria and developing the criteria weights; (2) aggregating performance and ranking of alternatives.

After selecting sets of alternatives and determining the most important criteria, the initial decision matrix *X* is developed:

$$X=\begin{array}{c} A_{1}\\ A_{2}\\ \ldots \\ A_{N} \end{array}\begin{array}{c} \begin{array}{cccc} C_{1} & C_{2} & \;\ldots \; & C_{M} \end{array}\\ \left[\begin{array}{cccc} x_{11} & x_{12} & \ldots \; & x_{1M}\\ x_{21} & x_{22} & \ldots \; & x_{2M}\\ \ldots & \ldots & \ldots \; & \ldots \\ x_{N1} & x_{N2} & \ldots \; & x_{NM} \end{array}\right] \end{array},$$

where $x_{ij}$ $(x_{ij}\geq 0$) is the value of *i*-th alternative on *j*-th criterion. Expert evaluations, results obtained from laboratory experiments, industrial measurements or computer simulations can be used to fill in matrix *X*.

The weighting coefficients of the criteria are described by vector $W=\left[\begin{array}{cccc} w_{1} & w_{2} & \ldots & w_{M} \end{array}\right]$, where $w_{j}$ ($0<w_{j}<1$) is the relative weight of the *j*-th criterion and $\sum_{j=1}^{M}w_{j}=1$.

During the first stage, the weights of criteria are calculated according to their importance for decision makers. Methods such as AHP, DEMATEL, Stepwise Weight Assessment Ratio Analysis (SWARA), entropy method, BWM or Full Consistency Method (FUCOM) can be employed. While the calculations in AHP and DEMATEL are based on matrix of pairwise comparisons, other methods require less input data. For example, for FUCOM, it is sufficient to set ranking of weights and ratios between adjacent coefficients.

During the second stage, the ranking of compared alternatives is performed by multi-attribute decision-making algorithms such as SAW, VIKOR, Complex Proportional Assessment (COPRAS), Additive Ratio Assessment (ARAS), TOPSIS, Weighted Aggregated Sum Product Assessment (WASPAS), Evaluation based on Distance from Average Solution (EDAS), Multi-Attributive Border approximation Area Comparison (MABAC), Combinative Distance-based Assessment (CODAS), Multi-Objective Optimization on the basis of Ratio Analysis (MOORA), TODIM, MARCOS or Range of Values (ROV).

The above-mentioned multi-criteria methods belong to two main groups, with additive weighted functions (SAW, WASPAS, MOORA, ROV) and according to the distance to the best and worst or average alternatives (TOPSIS, VIKOR, COPRAS, ARAS, EDAS, MABAC, CODAS, TODIM, MARCOS). The variety of multi-criteria methods allow for the different points of view of decision-makers to be taken into account when evaluating alternatives.

The ranking of alternatives is obtained after applying the preferred multi-criteria method. The alternative with the highest performance is the best choice among the alternatives set.

### 3.2. Conceptual Framework for IoT System Selection

An IoT system connects a multitude of devices in an ecosystem using different network and data protocols. A typical IoT system includes several main components:

- IoT devices—a set of connected sensors, appliances, vehicles, industrial robots;
- gateways—to link local devices network to Internet;
- network servers—to accept and transfer IoT data usually in cloud data centers;
- cloud applications—for IoT data processing;
- user interface—to visualize IoT data, track KPIs and send commands back to IoT devices.

The variety of IoT platforms and the many possible combinations of their features complicate the procedure of selecting the best alternative and underscore the necessity of a rigorous theoretical framework for the IoT platform selection problem [51].

Let $IoT\_Alternatives=\left\{A_{1},\;A_{2},\;\ldots ,\;A_{N}\right\}$ be a given set of systems and $IoT\_Criteria=\left\{C_{1},\;C_{2},\;\ldots ,\;C_{M}\right\}$ be a set of criteria. The features set can include technical, economic and environmental characteristics; for example, scalability, edge intelligence and support; key performance indicators and price model; energy consumption and the ability to generate renewable energy. Each alternative $A_{i}\in IoT\_Alternatives,\;i=\bar{1,N}$ corresponds to a subset of IoT_Criteria.

The problem is to rank the given systems according to their evaluations in a decision matrix, denoted as $Evaluations_{N\times M}$ for the defined set of criteria.

**Figure 2:**
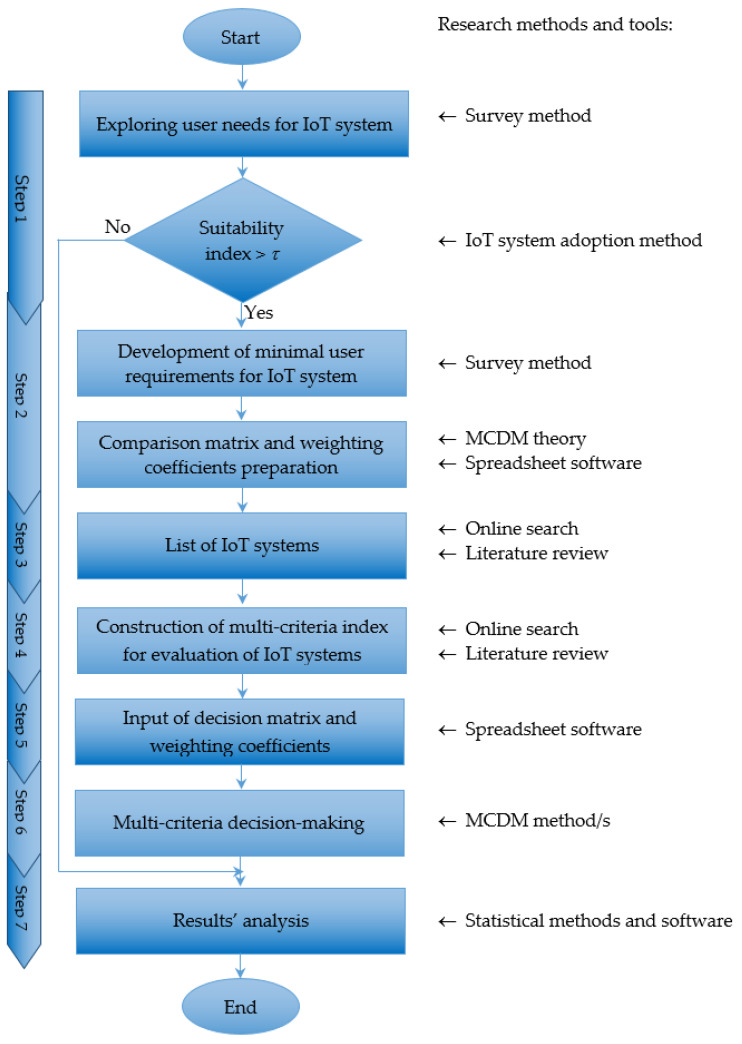
A flowchart of the proposed framework for IoT system selection.

Given that each IoT system could be characterized by using vague assessments for each criterion, the core of our new framework should be the fuzzy MCDM approach. The proposed conceptual framework consists of seven steps, as depicted in Figure 2.

Step 1. Exploring user’s IoT system needs

In the first stage of this step, in order to collect data about a company’s business model, we propose to apply the survey method. There are many questions that could be listed in the survey form; for example, needs (yes/no) for high availability, streaming data availability, extensive data support, offline functionality, real time analytics and visualization tools.

Next, in the second stage, a suitability index *τ* is calculated as a measure of company’s readiness for IoT deployment. If the index value obtained for a particular organization is larger than a predefined threshold, the company could be considered as suitable for IoT technology adoption, and the selection process can continue to Step 2. Otherwise, it should go to the end of the IoT platform selection process.

Step 2. Development of user requirements specification for IoT system

In order to collect data about consumer requirements, the survey method is used once again. The questionnaire consists of several question groups, corresponding to the various aspects of an autonomous company’s equipment (things, gateway, cloud, data analytics and user interface). At the end of this step, the minimal values of features of a preferred IoT platform are defined. Additionally, a matrix for the comparison of criteria importance is filled.

Remark: A team of experts takes part in Step 1 and Step 2 of the theoretical framework. These experts may be employees of the company or external specialists in the field of IoT.

Step 3. Search for a list of available IoT systems

In this step, a list of available IoT products (general purpose and/or industry-specific; open-source and/or proprietary) on the market that satisfy user’s requirements from Step 2 is obtained. To fill this list, an online data search and literature review could be employed.

Step 4. Design of multi-criteria system for IoT systems’ evaluation

In this step, a multi-criteria hierarchical evaluation index for IoT systems comparison is proposed. It encompasses different groups of indicators with specific relative weights depending on their importance for a company’s business processes. This composite index is flexible and allows for other groups and indicators to be considered for incorporation in it, depending on users’ preferences.

Step 5. Determination of decision matrix and preprocessing with calculation of weighting coefficients

First, based on data about the company’s industry and user requirements specification (Step 2), available datasets for IoT systems’ comparison (Step 3) and personalized multi-criteria evaluation system (Step 4), the corresponding assessments are filled in the decision matrix. In the case of Likert scale assessments, they could be transformed in intuitionistic fuzzy numbers (IFNs) using the 3-touple (Agree, Disagree, Neutral). The conversion is also possible for other advanced fuzzy sets such as Pythagorean (2013), picture (2013), Fermatean (2020) and other fuzzy sets. If there are categorical variables, they are converted in advance into linguistic variables. In the case that alternatives are evaluated by a group of experts, the decision matrix is filled after the arithmetic means aggregation of their assessments. In this way, the evaluations of each IoT system’s feature are calculated.

Second, the values of weighting coefficients are determined. The input data for calculations is the matrix of comparison of importance of IoT features by pairs (Step 2). In the case of a hierarchy of dimensions, the comparison should be provided for each hierarchical level from the top to the bottom. The weighting coefficients are calculated by using weight determination methods in crisp or fuzzy values.

Remark: In order to avoid a possible incongruity between some criteria, two different approaches can be applied. One of them includes some weight determination methods, such as AHP or the Analytic Network Process (ANP). These methods check the consistency of pairwise comparisons made by participants. The other approach is Inter-criteria Decision Analysis, which allows for removing any redundant criteria or objects from the original input data. Both approaches minimize discrepancies in participants’ opinions.

Step 6. Execution of multi-criteria decision-making methods

This step calculates the ranking of IoT systems using one or several MCDM methods with crisp or fuzzy values.

Step 7. Analysis of obtained results

Only the top-ranked alternatives from Step 6 are taken into consideration. Finally, decision-makers select the product that is the most appropriate for the company’s purposes.

At the end of the procedure, the IoT system with the greatest potential to enhance competitiveness will be deployed.

The advantages of the new framework are: (1) it implements a variety of (group) methods for weight determination and multi-criteria analysis and their combinations; (2) alternatives assessments could be expressed not only by real numbers, but also by a variety of fuzzy numbers or by fuzzy relations; (3) assessments can be made by a group of experts; (4) it is flexible and can be further extended to include new multi-criteria methods and types of assessments (such as advanced fuzzy sets).

### 3.3. New MABAC Modification for Intuitionistic Fuzzy Environment

The Multi-Attribute Border Approximation Area Comparison (MABAC) method is part of the MCDM group, and it determines similarity between each alternative and the best and worst value for each attribute using distance metrics. MABAC ranks the given opportunities according to their distance to the benchmarking values [52].

The classical MABAC method consists of six steps, described as follows:

Step 1. Input of the decision matrix and weighting coefficients.

Let $x_{ij}$ refers to the decision value related to the assessment of the *i*-th alternative against the *j*-th criteria in decision matrix Evaluations and $w_{j},\;j=\bar{1,M}$ are weighting coefficients of criteria.

Step 2. Normalization.

The normalized matrix $T={\left[t_{ij}\right]}_{N\times M}$ is calculated as:

$$t_{ij}=\{\;\begin{array}{c} \frac{x_{ij}-x_{j}{}^{min}}{x_{j}{}^{max}-x_{j}{}^{min}},\;j\in B;\\ \frac{x_{ij}-x_{j}{}^{max}}{x_{j}{}^{min}-x_{j}{}^{max}},\;j\in \mathbb{C}. \end{array}$$

where $x_{j}{}^{max}=\underset{j}{max}\left(x_{1},\;x_{2},\;\ldots ,\;x_{M}\right)$, $x_{j}{}^{min}=\underset{j}{min}\left(x_{1},\;x_{2},\;\ldots ,\;x_{M}\right)$, B denotes the set of maximizing criteria and ℂ is the group of minimizing criteria.

Step 3. Weighted matrix.

Let $V={\left[v_{ij}\right]}_{N\times M}$ be the weighted normalized decision matrix, where $v_{ij}$ refers to the weighted normalized decision value:

$$v_{ij}=w_{j}t_{ij}$$

Step 4. Matrix of border approximation area.

The border approximation area *G* of each criterion is defined as follows:

$$g_{j}=\overset{N}{\underset{i=1}{\prod }}v_{ij}^{1/N}.$$

Step 5. Matrix of distance to the border approximation area.

The matrix of distance to the border approximation area is calculated as follows:

$$q_{ij}=v_{ij}-g_{j},\;i=\bar{1,N},$$

where $q_{ij}$ is the distance to the border approximation area.

The belonging of alternative $A_{i}$ to the approximation area (G, $G^{+}$ or $G^{-}$) is determined on the basis of the following equation:

$$A_{i}\in \;\{\begin{array}{c} \begin{array}{c} G^{+},\;if\;q_{ij}>0\;\\ 0,\;if\;q_{ij}=0 \end{array}\\ G^{-},\;if\;q_{ij}<0 \end{array}$$

Step 6. Alternatives’ rank. The total distance of each alternative to the border approximation area is given by the next formula:

$$S_{i}=\overset{M}{\underset{j=1}{\sum }}q_{ij}.$$

The rank the alternatives is based on $S_{i}$ values, sorted in descending order [52].

We propose an intuitionistic version of the MABAC algorithm. Intuitionistic fuzzy sets (1986) are an extension of Zadeh’s fuzzy sets (1965) and more than thirty-five years of research reveals their potential to model the vagueness and ambiguity in real-world problems. With their three semantic components (degree of membership, degree of non-membership and hesitancy degree), intuitionistic fuzzy numbers are more expressive than classic fuzzy numbers. Besides that, the arithmetic operations with IFNs are relatively simple, compared to those of their advanced successors.

In case of intuitionistic fuzzy assessments of alternatives, the above-mentioned calculations are made according to the rules of intuitionistic fuzzy arithmetic.

An intuitionistic fuzzy number (IFN) is characterized by $\theta =\left(\mu_{\theta },\;\eta_{\theta }\right)$, where $\mu_{\theta }$ is degree of membership (truth), $\eta_{\theta }$ is degree of non-membership (falsity), $\pi_{\theta }=1-\mu_{\theta }-\eta_{\theta }$ is hesitancy degree and it holds $\mu_{\theta },\;\eta_{\theta }\in \left[0,1\right]$ and $0\leq \mu_{\theta }+\eta_{\theta }\leq 1$. Then,

(1) s(θ)=μ+μ(1−μ−η),

is said to be score function of an IFN θ [53].

Let $A=\left(\mu_{1},\;\eta_{1}\right)$ and $B=\left(\mu_{2},\;\eta_{2}\right)$ be two intuitionistic fuzzy numbers. The arithmetic operations with these intuitionistic fuzzy numbers are defined as follows:

$$\mathrm{Addition}:\;A+B=\left(\mu_{1}+\mu_{2}-\mu_{1}\mu_{2},\;\eta_{1}\eta_{2}\right)\;\mathrm{Subtraction}:\;A-B=\{\begin{array}{c} \langle \frac{\mu_{1}-\mu_{2}}{1-\mu_{2}},\;\frac{\eta_{1}}{\eta_{2}}\rangle ,\;if\;0\leq \;\frac{\eta_{1}}{\eta_{2}}\leq \frac{1-\mu_{1}}{1-\mu_{2}}\leq 1,\;\\ \langle 0,1\rangle ,\;\mathrm{otherwise}. \end{array}$$

$$\mathrm{Multiplication}:\;A\times B=\left(\mu_{1}\mu_{2},\eta_{1}+\eta_{2}-\eta_{1}\eta_{2}\right)$$

$$\mathrm{Division}:\;A\;/\;B=\{\begin{array}{c} \;\langle \frac{\mu_{1}}{\mu_{2}},\;\frac{\eta_{1}-\eta_{2}}{1-\eta_{2}}\rangle ,\;if\;0\leq \;\frac{\mu_{1}}{\mu_{2}}\leq \frac{1-\eta_{1}}{1-\eta_{2}}\leq 1,\;\\ \langle 0,1\rangle ,\;\mathrm{otherwise}. \end{array}$$

The λ times of A is given by the next rule [54]:

$$\lambda A=\langle 1-{\left(1-\mu_{1}\right)}^{\lambda },\eta_{1}{}^{\lambda }\rangle .$$

In the new MABAC version, in order to calculate the distance to the border approximation area (Step 5) in intuitionistic fuzzy environment, we employ a new similarity measure $S_{GC}^{1}$ between $A_{p}$ and $A_{q}$ alternatives, assessed by intuitionistic fuzzy sets according to the following formula [55]:

$$S_{GC}^{1}\left(A_{p},A_{q}\right)=1-\sqrt{\frac{1}{12M}\overset{M}{\underset{j=1}{\sum }}\left[(D_{\mu j}{}^{2}+D_{\eta j}{}^{2})D_{\pi j}{}^{2}+2\left(D_{min_{j}}{}^{2}+D_{max_{j}}{}^{2}\right)\right]}$$

where $A_{p}=\left\{\langle \mu_{pj},\eta_{pj},\;j=\bar{1,M}\rangle \right\}$, $A_{q}=\left\{\langle \mu_{qj},\eta_{qj},j=\bar{1,M}\rangle \right\}$, $D_{\mu_{pj}}=\mu_{pj}-\mu_{qj}$, $D_{\eta_{pj}}=\eta_{pj}-\eta_{qj},$ $\pi_{pj}=1-\mu_{pj}-\eta_{pj}$, $\pi_{qj}=1-\mu_{qj}-\eta_{qj}$, $D_{\pi_{j}}=2-\left|\pi_{pj}-\pi_{qj}\right|,$ $D_{min_{j}}=min\left[\mu_{pj},\mu_{qj}\right]-min\left[\mu_{qj},\eta_{pj}\right]$ and $D_{max_{j}}=max\left[\mu_{pj},\eta_{qj}\right]-max\left[\mu_{qj},\eta_{pj}\right]$.

This distance formula employs intuitionistic fuzzy numbers, which means that the choice is based on more accurate estimation. The assessments’ representation in IFNs comprises a pair of semantically opposite values—membership (truth) and non-membership (falsity) degree. The advantage of utilized formula is that the similarity between intuitionistic fuzzy sets is calculated in three-dimensional space, using additionally the third membership degree–hesitancy. The novelty of this formula is that here the similarity depends on the difference between the maximum and the minimum of the cross-evaluation factor.

In order to assess the performance of IoT platforms objectively, we propose a new modification of MABAC for intuitionistic fuzzy environment. Intuitionistic uncertainty and hesitancy degrees account for differences in decision makers’ estimates more accurately than classical fuzzy numbers. In addition, the existing intuitionistic aggregating operators and distance metrics successfully combine individual estimates into a complex measure of the quality of compared alternatives. The main disadvantage of the proposed method lies in the higher time complexity of its similarity formula compared to the classical MABAC. However, the increase in computing time is compensated by more accurate measurement of the distance between the given IoT systems.

## 4. Verification of MCDM Conceptual Framework

In this section, we apply the new methodological framework to solve a practical problem for IoT platform selection. Then, the IoT systems are estimated by using new MABAC modification with intuitionistic fuzzy values. Finally, we discuss the performance of the proposed enhanced multi-criteria method.

### 4.1. Practical Example

Let *AF* be a randomly selected company exposed to an IoT platform selection problem. The benefits of IoT for remote, distributed and continuous control over traditional software are numerous. The problem is how to find the best IoT-based system for the particular company.

Step 1. A team of experts from company *AF* fill in the questionnaire about their IoT system’s requirements [51]. Let the execution of Step 1 of the proposed framework show that the company’s *AF* suitability index is greater than the given threshold.

Step 2. By using a survey method, the minimal user requirements for IoT platform are determined and a comparison matrix of weighting coefficients is filled in. Respondents evaluate the IoT platforms’ features using five-point Likert scale from “totally agree” (corresponding to 5) to “totally disagree” (corresponding to 1).

Step 3. In this illustrative example, we utilize an IoT platforms’ dataset, collected from Ullah et al. [51]. By using online search and literature review the list of IoT platforms is determined. The list consists of five IoT-based products (*A*_1_, *A*_2_, …, *A*_5_). The IoT-based platforms are as follows: *A*_1_—AWS IoT, *A*_2_—MS Azure IoT, *A*_3_—Google Cloud IoT, *A*_4_—IBM Watson IoT, and *A*_5_—Oracle IoT.

Step 4. In this step, an evaluation index *C* is constructed, $C=\left[C_{j}\right],\;j=\bar{1,21}$. The assessments of alternatives by criteria are obtained by survey of the experts’ team. Each criterion represents a product feature of IoT platforms: *C*_1_—scalability, *C*_2_—flexibility, *C*_3_—data analytics, *C*_4_—disaster recovery, *C*_5_—stability, *C*_6_—security, *C*_7_—data ownership, *C*_8_—protocol support, *C*_9_—system performance, *C*_10_—time to market, *C*_11_—legacy architecture, *C*_12_—attractive interface, *C*_13_—pricing model, *C*_14_—cloud ownership, *C*_15_—interoperability, *C*_16_—application environment, *C*_17_—hybrid cloud, *C*_18_—platform migration, *C*_19_—previous experience, *C*_20_—Edge intelligence, and *C*_21_—bandwidth [51].

Step 5. Input of decision matrix and criteria weights

The decision matrix values are converted into intuitionistic fuzzy numbers. The importance assessments are interpreted as intuitionistic fuzzy numbers and after that, they are converted into crisp values by using the score function.

Step 6. By using preferred MCDM methods, the scores and rankings of given IoT platforms are calculated.

Step 7. Results’ analysis.

In the analysis of results, only IoT platforms that have been top ranked with preferred MCMD methods are left. In this step, decision-makers select the most suitable IoT product.

The detailed calculations from Step 5, Step 6 (IFNs MABAC) and Step 7 are available in the next section.

### 4.2. MCDM Using IFSs MABAC

Step 1. Input of decision matrix and weighting coefficients

The experts’ estimates (linguistic variables) and the importance of criteria (as agreed, disagreed and neutral percentages) according to questionnaire answers are filled in Table 2. The linguistic variables are converted into crisp numbers using a simple correspondence rule. The obtained values are written in the decision matrix $Evaluations={\left[x_{ij}\right]}_{5\times 21}$.

Step 2. Normalization.

The normalized matrix $T={\left[t_{ij}\right]}_{5\times 21}$ is calculated as:

$$t_{ij}=\{\;\begin{array}{c} \frac{x_{ij}-x_{j}{}^{min}}{x_{j}{}^{max}-x_{j}{}^{min}},\;j\in B;\\ \frac{x_{ij}-x_{j}{}^{max}}{x_{j}{}^{min}-x_{j}{}^{max}},\;j\in \mathbb{C}. \end{array}$$

In our case, the criteria are only maximizing. Missing values are replaced with mean average value of the corresponding criteria.

The importance of each factor is calculated according to the score function formula, where agreed, disagreed and neutral percentages correspond to positive, negative and hesitance memberships’ values, respectively. The final weights $w_{j}$, *j* = $\bar{1,21}$ are normalized such that:

$$\overset{21}{\underset{j=1}{\sum }}w_{j}=1.$$

The obtained weighting coefficients are as follows: $w_{1}$ = $w_{2}$ = … = $w_{9}$ *=* 0.051, $w_{10}$ = $w_{16}$ = 0.047, $w_{11}$ = $w_{12}$ = 0.05, $w_{13}$ *=* $w_{14}$ = 0.049, $w_{15}$ = 0.046, $w_{17}$ = 0.045, $w_{18}$ = $w_{19}$ = 0.042, and $w_{20}$ = $w_{21}$ = 0.038.

The obtained crisp values after normalization are shown in Table 3.

Step 3. Decision matrix (IFNs) and its normalization

The crisp values of normalized matrix are converted into intuitionistic fuzzy numbers by Visalakshi et al. formula [56]

$$\mu =1-{\left(1-\bar{\mu }\right)}^{\lambda }\;\eta ={\left(1-\bar{\mu }\right)}^{\lambda \left(\lambda +1\right)},$$

where $\bar{\mu }$ is the crisp value and *λ* ∈ [0, 1], here *λ* = 0.5 (Table 4).

The weighted normalized matrix $V={\left[v_{ij}\right]}_{5\times 21}$ is calculated by using formula for λ times (Table 5):

$$v_{ij}=w_{j}t_{ij}$$

Step 4. Matrix of border approximation area.

The border approximation area *G* of each criterion is calculated as follows:

$$g_{j}=\overset{21}{\underset{i=1}{\prod }}v_{ij}^{1/21}=\langle \left[\overset{21}{\underset{i=1}{\prod }}{\left(\mu_{ij}\right)}^{1/21}\right],\left[1-\overset{21}{\underset{i=1}{\prod }}{\left(1-\eta_{ij}\right)}^{1/21}\right]\rangle .$$

The results are shown in Table 6.

Step 5. Matrix of distance to the border approximation area.

The values of distance matrix to the border approximation area

$$q_{ij}=v_{ij}-g_{j},\;i=\bar{1,21}$$

are calculated by using $S_{GC}^{1}$ formula (Table 7).

The belonging of alternative $A_{i}$ to the approximation area (G, $G^{+}$ or $G^{-}$) is determined on the basis of the following equation:

$$A_{i}\in \;\{\begin{array}{c} \begin{array}{c} G^{+},\;if\;q_{ij}>0\;\\ 0,\;if\;q_{ij}=0 \end{array}\\ G^{-},\;if\;q_{ij}<0 \end{array}$$

Step 6. Alternatives’ rank.

The total distance of each alternative to the border approximation area is obtained by the next formula:

$$S_{i}=\overset{5}{\underset{j=1}{\sum }}q_{ij}.$$

The rank the alternatives is based on $S_{i}$ values, sorted in ascending order (Table 8).

The obtained ranking by using new intuitionistic fuzzy MABAC is as follows:

$$S_{GC}^{1}\;\mathrm{MABAC}:\;A_{1}\;≻\;A_{2}\;≻\;A_{3}\;\approx \;A_{5}\;≻\;A_{4},$$

i.e., *A*_1_ platform (AWS IoT) is the most suitable for *AF* company according to the given requirements.

Step 7. In order to check the reliability of proposed model and the consistency of the results produced by intuitionistic fuzzy assessments, the same problem is solved by using crisp SAW, crisp MABAC and IFNs MABAC with *L*_2_ discrimination measure [57] (Table 9).

### 4.3. Analysis of Obtained Results

The obtained rankings are almost identical or similar to the $S_{GC}^{1}$ MABAC result:

Crisp SAW: A_1_ ≻ A_2_ ≻ A_3_ ≻ A_5_ ≻ A_4_;

Crisp MABAC: A_2_ ≻ A_1_ ≻ A_3_ ≻ A_4_ ≻ A_5__;_

IFNs *L*_2_ MABAC: A_2_ ≻ A_3_ ≻ A_1_ ≻ A_4_ ≻ A_5_;

Spearman’s rank correlation coefficient is applied as a similarity measure between classical SAW and each other MCDM methods’ rankings. Spearman’s coefficients indicates high degrees of closeness of obtained rankings—0.8 (crisp MABAC), 0.6 (*L*_2_ MABAC) and 1.0 ($S_{GC}^{1}$ MABAC). This means that the proposed intuitionistic fuzzy model is reliable and could be applied for MCDM.

The analysis also shows that two groups of IoT platforms can be distinguished in the obtained crisp and fuzzy MCDM rankings:

Group 1. IoT platforms with high assessments—A_1_, A_2_ and A_3_;

Group 2. IoT platforms with relative low assessments—A_4_ and A_5_.

The highest experts’ ratings (crisp SAW and $S_{GC}^{1}$ MABAC) of alternative *A*_1_ (AWS IoT) assign it to the leading group, while alternatives *A*_4_ (IBM Watson IoT) and *A*_5_ (Oracle IoT) falls into the second part of the ranking (crisp SAW, crisp MABAC, *L*_2_ MABAC and $S_{GC}^{1}$ MABAC). One possible reason for the worse ranking of IBM Watson IoT and Oracle IoT is that both platforms are more encapsulated in their company’s ecosystems than other three platforms. The good performance of *A*_1_ and *A*_2_ correspond to the GMQ’2021 assertion, that AWS IoT and Azure IoT are “challenger” and “leader” respectively among the best IIoT platforms. Therefore, it can be concluded that the proposed algorithm is robust and reliable, because the obtained ranking meets the customers’ preferences in company *AF*. Another advantage of the new multi-criteria method in comparison with optimization and machine learning methods is that the results are easily explainable.

The utilization of $S_{GC}^{1}$ MABAC not only led to a better treatment of subjective experts’ opinions, but also to a reduction in time complexity in comparison with recently created analog *L*_2_ MABAC. By using the new distance formula between IFNs in MABAC, a good balance between low computational complexity, simplicity of decision-making model and its effectiveness was achieved.

The comparison with results obtained from similar previous studies shows the following:

- Despite the large number of methodologies for IoT system selection, only several studies investigate and analyze more than two MCDM methods [17,18,22,25] or employ the fuzzy approach [15,18,24,25,26].
- Some of the researchers compare only specialized IoT platforms or particular elements of IoT infrastructure [16,20,25].
- In some studies, a practical example for the ranking of IoT alternatives is missing [18,19,22].
- The obtained rankings of IoT platforms are almost identical to those obtained by Kondratenko et al. and Chakraborty (AWS, Azure, Google, IBM) [24,26], Lin et al. (AWS, Azure, IBM) [15] and Youssef (AWS, Azure, Google) [21].

The proposed framework systematizes common rules and procedures for group multi-criteria selection of IoT platforms. The new framework ensures feasible solution using users’ needs and avoiding subjectivism in experts’ opinions. It facilitates the construction of complex indices for systems evaluation, including technical specifications, key performance indicators and metrics for the sustainability of IoT ecosystems. Furthermore, it is flexible and timesaving, reducing the possibility of errors while preparing relevant input data for each step of the decision-making process. Unlike previous similar studies, the new framework could implement multi-criteria analysis in different fuzzy environments (classical and intuitionistic).

## 5. Conclusions

The process of determining the best suitable IoT platform depends on many factors, (peculiarities of business processes and legacy systems, users’ preferences, vendor’s profile to name a few), i.e., it is in fact a multi-criteria decision-making problem. This study outlines a multi-criteria framework for IoT platform selection in a fuzzy environment. In the proposed framework, a new modification of Multi-Attribute Border approximation Area Comparison (MABAC) method with specific similarity measure via intuitionistic fuzzy values has been presented as a decision analysis method. The new technique is more precise than existing crisp and fuzzy analogues, as its (1) calculations include the three semantic components of intuitionistic fuzzy numbers and (2) distance formula takes into account the relationship between cross-evaluation of membership (truth) degrees as addition to the difference between main intuitionistic components. The effectiveness of the new decision-making framework has been verified through an illustrative example of ranking IoT platforms.

The new framework automates the process of ranking of IoT systems and has several advantages:

(1) The comparison is based on a complex index that covers technological and business requirements of a team of experts and consumer preferences;
(2) MCDM methods have the capability to handle vague and uncertain estimates of both cost and beneficial criteria via fuzzy values;
(3) The proposed evaluation system is flexible. It can be further extended with additional decision-making algorithms and adapted to other organizations or sectors;
(4) The weighting coefficients and decision matrix are determined by a group of experts familiar with the company’s business processes and IoT technology.

The advantage of the proposed fuzzy modification of MABAC method is that the distance between compared alternatives is calculated by an improved formula for similarity in intuitionistic environment:

- In addition to membership and non-membership parameters, a hesitancy degree is included;
- The difference of maximum of the cross-evaluation factor and the difference of minimum of the cross-evaluation factor also participates in the calculation.

The validity of the new framework is demonstrated using a practical example for the selection of IoT platforms. The problem is to find the best ranking alternative from five IoT platforms (AWS IoT Core, Microsoft Azure IoT, Google Cloud IoT Core, IBM Watson IoT and Oracle IoT) according to twenty-one criteria for comparison. The analysis of obtained results shows that the proposed methodology is reliable and correctly reflects user’s requirements.

In the future, the theoretical framework will be improved by aggregating several rankings obtained through different multi-criteria methods using meta-methods. Additionally, the proposed mechanisms for the ranking of IoT alternatives will be expanded to address uncertainty of estimates with different types of fuzzy sets (for example, type-2 fuzzy numbers and spherical fuzzy numbers). We also have a plan to develop new hybrid methods for IoT systems’ evaluation combining weights determination algorithms with multi-criteria decision-making methods.

## Figures and Tables

**Table 1:** A comparison of the most widely used IoT platforms.

Platform Feature	AWS	MS Azure	Google Cloud	IBM Watson	Oracle
IoT Core Functions	Connectivity, authentication, rules engine, Developer mode, Edge SDK	Connectivity, authentication, device management, device monitoring, IoT Edge SDK	Connectivity, authentication, device management	Connectivity, authentication, device management, Edge computing	Connectivity, authentication, device management, Edge computing
Edge Computing Solutions	Free RTOS ^1^	IoT Edge	Edge TPU ^2^ chip	IBM Edge app manager	IoT Edge
Data Protocols	HTTP, MQTT, WebSockets	HTTP, MQTT, AMQP over WebSockets	HTTP, MQTT	HTTP, MQTT	HTTP, MQTT
Data Processing and Analytics	S3, Kinesis, Lambda, Dynamo DB, Elastic Search	Stream Analytics, Azure Blob, Notification, Power BI	Dataflow, Big Data, Big Query, Coding Service Engine	Activity Tracker, Log Analysis, Monitoring, Pak for Watson AIOps	Business Intelligence Cloud, Analytics Cloud, and Mobile Cloud services

^1^ RTOS—Real-time Operating Systems. ^2^ TPU—Tensor Processing Unit.

**Table 2:** Experts’ estimates and the importance of criteria for IoT platforms assessment (linguistic variables).

Criteria	A1	A2	A3	A4	A5	Importance (%)		
						Agree	Neutral	Disagree
C1	yes	yes	yes	yes	yes	93%	7%	0%
C2	yes	-	yes	-	yes	93%	7%	0%
C3	yes	yes	yes	yes	yes	93%	7%	0%
C4	yes	yes	no	no	no	93%	7%	0%
C5	yes	yes	yes	-	-	93%	7%	0%
C6	high	high	high	high	high	93%	7%	0%
C7	-	yes	-	-	-	93%	7%	0%
C8	yes	yes	-	yes	yes	93%	7%	0%
C9	yes	-	yes	yes	-	93%	7%	0%
C10	yes	yes	-	-	yes	86%	7%	7%
C11	yes	-	-	-	yes	86%	14%	0%
C12	yes	yes	-	no	-	86%	14%	0%
C13	bad	bad	good	-	-	79%	21%	0%
C14	yes	yes	yes	-	yes	79%	21%	0%
C15	yes	-	-	-	yes	79%	14%	7%
C16	yes	yes	yes	yes	yes	71%	29%	0%
C17	yes	yes	-	-	-	64%	36%	0%
C18	yes	yes	-	-	-	64%	29%	7%
C19	yes	yes	-	-	-	64%	29%	7%
C20	yes	yes	yes	-	yes	57%	29%	14%
C21	-	-	good	-	-	57%	29%	14%

Remark: This table shows the transposed input matrix. Reprinted with permission from Ref. [51]. 2020, IEEE.

**Table 3:** Normalized decision matrix *T* and weighted coefficients $w_{j}$ (crisp values).

	A1	A2	A3	A4	A5	$w_{j}$
C1	0.950	0.950	0.950	0.950	0.950	0.051
C2	0.950	0.570	0.950	0.570	0.950	0.051
C3	0.950	0.950	0.950	0.950	0.950	0.051
C4	0.950	0.950	0.050	0.050	0.050	0.051
C5	0.950	0.950	0.950	0.570	0.570	0.051
C6	0.950	0.950	0.950	0.950	0.950	0.051
C7	0.191	0.950	0.191	0.191	0.191	0.051
C8	0.950	0.950	0.760	0.950	0.950	0.051
C9	0.950	0.570	0.950	0.950	0.570	0.051
C10	0.950	0.950	0.570	0.570	0.950	0.047
C11	0.950	0.381	0.381	0.381	0.950	0.050
C12	0.950	0.950	0.390	0.050	0.390	0.050
C13	0.050	0.050	0.950	0.210	0.210	0.049
C14	0.950	0.950	0.950	0.760	0.950	0.049
C15	0.950	0.381	0.381	0.381	0.950	0.046
C16	0.950	0.950	0.950	0.950	0.950	0.047
C17	0.950	0.950	0.381	0.381	0.381	0.045
C18	0.950	0.950	0.381	0.381	0.381	0.042
C19	0.950	0.950	0.381	0.381	0.381	0.042
C20	0.950	0.950	0.950	0.619	0.950	0.038
C21	0.191	0.191	0.950	0.191	0.191	0.038

**Table 4:** The decision matrix *T* (IFNs).

	A1		A2		A3		A4		A5	
C1	0.776	0.106	0.776	0.106	0.776	0.106	0.776	0.106	0.776	0.106
C2	0.776	0.106	0.001	0.999	0.776	0.106	0.001	0.999	0.776	0.106
C3	0.776	0.106	0.776	0.106	0.776	0.106	0.776	0.106	0.776	0.106
C4	0.776	0.106	0.776	0.106	0.025	0.962	0.025	0.962	0.025	0.962
C5	0.776	0.106	0.776	0.106	0.776	0.106	0.001	0.999	0.001	0.999
C6	0.776	0.106	0.776	0.106	0.776	0.106	0.776	0.106	0.776	0.106
C7	0.001	0.999	0.776	0.106	0.001	0.999	0.001	0.999	0.001	0.999
C8	0.776	0.106	0.776	0.106	0.001	0.999	0.776	0.106	0.776	0.106
C9	0.776	0.106	0.001	0.999	0.776	0.106	0.776	0.106	0.001	0.999
C10	0.776	0.106	0.776	0.106	0.001	0.999	0.001	0.999	0.776	0.106
C11	0.776	0.106	0.001	0.999	0.001	0.999	0.001	0.999	0.776	0.106
C12	0.776	0.106	0.776	0.106	0.001	0.999	0.025	0.962	0.001	0.999
C13	0.025	0.962	0.025	0.962	0.776	0.106	0.001	0.999	0.001	0.999
C14	0.776	0.106	0.776	0.106	0.776	0.106	0.001	0.999	0.776	0.106
C15	0.776	0.106	0.001	0.999	0.001	0.999	0.001	0.999	0.776	0.106
C16	0.776	0.106	0.776	0.106	0.776	0.106	0.776	0.106	0.776	0.106
C17	0.776	0.106	0.776	0.106	0.001	0.999	0.001	0.999	0.001	0.999
C18	0.776	0.106	0.776	0.106	0.001	0.999	0.001	0.999	0.001	0.999
C19	0.776	0.106	0.776	0.106	0.001	0.999	0.001	0.999	0.001	0.999
C20	0.776	0.106	0.776	0.106	0.776	0.106	0.001	0.999	0.776	0.106
C21	0.001	0.999	0.001	0.999	0.776	0.106	0.001	0.999	0.001	0.999

**Table 5:** The weighted decision matrix *V* (IFNs).

	A1		A2		A3		A4		A5	
C1	0.073	0.892	0.073	0.892	0.073	0.892	0.073	0.892	0.073	0.892
C2	0.073	0.892	0.000	1.000	0.073	0.892	0.000	1.000	0.073	0.892
C3	0.073	0.892	0.073	0.892	0.073	0.892	0.073	0.892	0.073	0.892
C4	0.073	0.892	0.073	0.892	0.001	0.998	0.001	0.998	0.001	0.998
C5	0.073	0.892	0.073	0.892	0.073	0.892	0.000	1.000	0.000	1.000
C6	0.073	0.892	0.073	0.892	0.073	0.892	0.073	0.892	0.073	0.892
C7	0.000	1.000	0.073	0.892	0.000	1.000	0.000	1.000	0.000	1.000
C8	0.073	0.892	0.073	0.892	0.000	1.000	0.073	0.892	0.073	0.892
C9	0.073	0.892	0.000	1.000	0.073	0.892	0.073	0.892	0.000	1.000
C10	0.068	0.900	0.068	0.900	0.000	1.000	0.000	1.000	0.068	0.900
C11	0.072	0.893	0.000	1.000	0.000	1.000	0.000	1.000	0.072	0.893
C12	0.072	0.893	0.072	0.893	0.000	1.000	0.001	0.998	0.000	1.000
C13	0.001	0.998	0.001	0.998	0.071	0.896	0.000	1.000	0.000	1.000
C14	0.071	0.896	0.071	0.896	0.071	0.896	0.000	1.000	0.071	0.896
C15	0.067	0.902	0.000	1.000	0.000	1.000	0.000	1.000	0.067	0.902
C16	0.068	0.900	0.068	0.900	0.068	0.900	0.068	0.900	0.068	0.900
C17	0.064	0.905	0.064	0.905	0.000	1.000	0.000	1.000	0.000	1.000
C18	0.061	0.910	0.061	0.910	0.000	1.000	0.000	1.000	0.000	1.000
C19	0.061	0.910	0.061	0.910	0.000	1.000	0.000	1.000	0.000	1.000
C20	0.055	0.919	0.055	0.919	0.055	0.919	0.000	1.000	0.055	0.919
C21	0.000	1.000	0.000	1.000	0.055	0.919	0.000	1.000	0.000	1.000

**Table 6:** Calculations and the obtained matrix of border approximation area *G*.

	A1		A2		A3		A4		A5		G	
C1	0.593	0.641	0.593	0.641	0.593	0.641	0.593	0.641	0.593	0.641	0.073	0.892
C2	0.593	0.641	0.121	0.131	0.593	0.641	0.121	0.131	0.593	0.641	0.003	0.996
C3	0.593	0.641	0.593	0.641	0.593	0.641	0.593	0.641	0.593	0.641	0.073	0.892
C4	0.593	0.641	0.593	0.641	0.265	0.287	0.265	0.287	0.265	0.287	0.007	0.990
C5	0.593	0.641	0.593	0.641	0.593	0.641	0.121	0.131	0.121	0.131	0.003	0.996
C6	0.593	0.641	0.593	0.641	0.593	0.641	0.593	0.641	0.593	0.641	0.073	0.892
C7	0.121	0.131	0.593	0.641	0.121	0.131	0.121	0.131	0.121	0.131	0.000	1.000
C8	0.593	0.641	0.593	0.641	0.121	0.131	0.593	0.641	0.593	0.641	0.015	0.978
C9	0.593	0.641	0.121	0.131	0.593	0.641	0.593	0.641	0.121	0.131	0.003	0.996
C10	0.584	0.631	0.584	0.631	0.119	0.129	0.119	0.129	0.584	0.631	0.003	0.996
C11	0.591	0.639	0.120	0.130	0.120	0.130	0.120	0.130	0.591	0.639	0.001	0.999
C12	0.591	0.639	0.591	0.639	0.120	0.130	0.264	0.286	0.120	0.130	0.001	0.998
C13	0.263	0.285	0.263	0.285	0.589	0.636	0.120	0.130	0.120	0.130	0.001	0.999
C14	0.589	0.636	0.589	0.636	0.589	0.636	0.120	0.130	0.589	0.636	0.014	0.979
C15	0.582	0.629	0.118	0.128	0.118	0.128	0.118	0.128	0.582	0.629	0.001	0.999
C16	0.584	0.631	0.584	0.631	0.584	0.631	0.584	0.631	0.584	0.631	0.068	0.900
C17	0.578	0.625	0.578	0.625	0.117	0.127	0.117	0.127	0.117	0.127	0.001	0.999
C18	0.572	0.618	0.572	0.618	0.116	0.126	0.116	0.126	0.116	0.126	0.001	0.999
C19	0.572	0.618	0.572	0.618	0.116	0.126	0.116	0.126	0.116	0.126	0.001	0.999
C20	0.559	0.605	0.559	0.605	0.559	0.605	0.113	0.123	0.559	0.605	0.011	0.984
C21	0.113	0.123	0.113	0.123	0.559	0.605	0.113	0.123	0.113	0.123	0.000	1.000

**Table 7:** The matrix of distances to the border approximation area *Q*.

	A1	A2	A3	A4	A5
C1	1.000	1.000	1.000	1.000	1.000
C2	0.975	0.999	0.975	0.999	0.975
C3	1.000	1.000	1.000	1.000	1.000
C4	0.976	0.976	0.998	0.998	0.998
C5	0.975	0.975	0.975	0.999	0.999
C6	1.000	1.000	1.000	1.000	1.000
C7	1.000	0.974	1.000	1.000	1.000
C8	0.979	0.979	0.995	0.979	0.979
C9	0.975	0.999	0.975	0.975	0.999
C10	0.977	0.977	0.999	0.999	0.977
C11	0.974	1.000	1.000	1.000	0.974
C12	0.974	0.974	1.000	1.000	1.000
C13	1.000	1.000	0.975	1.000	1.000
C14	0.980	0.980	0.980	0.995	0.980
C15	0.976	1.000	1.000	1.000	0.976
C16	1.000	1.000	1.000	1.000	1.000
C17	0.977	0.977	1.000	1.000	1.000
C18	0.978	0.978	1.000	1.000	1.000
C19	0.978	0.978	1.000	1.000	1.000
C20	0.984	0.984	0.984	0.996	0.984
C21	1.000	1.000	0.980	1.000	1.000

**Table 8:** The overall alternative scores and their corresponding ranking–new MABAC (IFNs).

	$S_{GC}^{1}\;\mathrm{MABAC}$	
	Score	Rank
A_1_	0.015	1
A_2_	0.012	2
A_3_	0.008	3
A_4_	0.003	5
A_5_	0.008	4

**Table 9:** The overall alternative scores and their corresponding rankings—crisp SAW, crisp MABAC and IFNs *L*_2_ MABAC.

	Crisp SAW		Crisp MABAC		*L*_2_ MABAC	
	Score	Rank	Score	Rank	Score	Rank
A_1_	0.839	1	0.375	2	0.110	3
A_2_	0.784	2	0.379	1	0.115	1
A_3_	0.682	3	0.344	3	0.113	2
A_4_	0.549	5	0.303	4	0.103	4
A_5_	0.661	4	0.295	5	0.102	5

## Data Availability

Not applicable.
